# Application of Felid Hair for Non-Invasive Tracking of Animal Reproductive Status and Adrenal Activity

**DOI:** 10.3390/ani12202792

**Published:** 2022-10-16

**Authors:** Sergey V. Naidenko, Galina S. Alekseeva, Polina S. Klyuchnikova, Mariya N. Erofeeva

**Affiliations:** Department of Behavior and Behavioral Ecology of Mammals, Severtsov Institute of Ecology and Evolution, Russian Academy of Sciences, 119071 Moscow, Russia

**Keywords:** testosterone, cortisol, hormone monitoring, caracal, seasonality, lynx, domestic cat

## Abstract

**Simple Summary:**

Hair can be a useful matrix to examine the hormonal status of an animal, although it is difficult to correlate the results to a specific time point. We measured seasonal changes in cortisol and testosterone levels in four feline species with different breeding strategies. Both hormones showed annual dynamic fluctuations, which coincided with the reproductive traits of the studied species. Seasonal increases in testosterone were observed in three seasonal breeders (lynx, Amur wildcat and domestic cat) but not in caracal, an aseasonal species, which sustained high testosterone levels over the whole year. Sexual differences in testosterone level were found for these species only during the mating season, when testosterone levels were higher in males than in females. Cortisol levels increased during the mating period, and in caracal an increase in cortisol was associated with the transfer of animals to smaller cages.

**Abstract:**

Hair can be a useful matrix to examine the hormonal status of an animal, although it is difficult to correlate the results to a specific time point. The aim of this study was to evaluate seasonal changes in cortisol and testosterone levels in the hair of four feline species (lynx, *Lynx lynx*, *n* = 8; Amur wildcat, *Prionailurus bengalensis euptilurus*, *n* = 8; caracal, *Caracal caracal*, *n* = 6 and domestic cat, Felis catus, *n* = 17) with different breeding strategies. Animals of both sexes were sampled over the year, once per season (every three months), and the concentrations of hair testosterone and cortisol were measured by EIA. Both hormones showed annual dynamic changes, which coincided with the reproductive seasonality of the studied species. Sexual differences in testosterone level were found only during the mating season (spring for lynx and Amur wildcats, spring–summer for domestic cats), when testosterone levels were higher in males than in females. Cortisol levels were higher in males than in females in domestic cats and Amur wildcat, but also only during the mating season. Seasonal increases in testosterone were observed in three seasonal breeders (lynx, Amur wildcat and domestic cat) but not in caracal, which had high testosterone levels over the whole year. In lynx and Amur wildcat, it decreased sharply in the summer. Cortisol levels increased during the mating period in domestic cat males and lynx of both sexes; in caracal, an increase in cortisol was related to the transfer of animals to smaller winter cages. Measurements of steroids in hair can provide a reliable method for evaluating the reproductive status and the activity of the hypothalamus–pituitary–adrenal axis in several felid species.

## 1. Introduction

The assessment of hormonal status in individual animals has been carried out in recent decades, not only in the blood [1] and organs [2] of animals but in a variety of excreta (feces [3], urine [4]), bodily fluids (saliva [5], milk [6]) and hair [7]. The latter evaluation method has been used for a long time in competitive sports to determine the presence of anabolic steroids [8,9]. Validation of measurements of steroid hormone concentrations in hair [10,11] is carried out more rarely than in excreta; however, in recent years, this analysis has become more and more routine [11,12,13]. It is believed that native hormones are present in the hair, as well as their metabolites [12].

Unlike many other substrates, it is rather difficult to estimate the time interval [11], which corresponds to the concentration of the hormone in the hair, in particular, how quickly hormones will enter the hair after circulating in the blood and what time interval (from weeks to months) is representative of bio-accumulation of hormones in the sample. The results in various species are somewhat inconsistent. It is believed that the level of hormones in the long hair of elephants (tail) [14] or orangutans [15] can reflect rather large time intervals, as in pinniped whiskers [16] or cetaceans baleen [17]. At the same time, the work carried out on adrenalectomized rats [18], followed by regular administration of corticosterone, showed that the level of corticosterone in their hair began to increase two weeks after the start of injections and began to decrease two weeks after they ended, thus reflecting a rather short life span of this hormone in animals. The correlations of the level of hair cortisol with the feces [19] or saliva [20] cortisol concentrations suggest that steroid hormones become present in hairs soon after their level increases in the blood stream [12]. 

The measurement of hair hormone levels has also been used for carnivores [10], to estimate interspecific [21] and intraspecific [22] differences in felines, and sometimes with large datasets such as those in bears [7,23,24], indicating that the determined level of the hormone (cortisol) characterizes the physiological state of animals quite well. The changes in hair glucocorticoids (both cortisol and corticosterone [25]) may be attributed to the effect of age and pregnancy, body region, sex and season of year, but these results are not very consistent [11,13].

However, despite the increased use of the method in recent years, there are not many studies with reliable validation proving the adequacy of its application [11,12,13,19]. It is likely that for different systematic groups of animals (rodents, proboscideans, carnivores) there are differences in the deposition of hormones in the hair and their storage time; therefore, for each group of animals, it is important to provide what information can be obtained by measuring the level of steroid hormones in the hair of animals. In domestic cats, the hair cortisol level correlates with the fecal cortisol that made this method reliable for the measurements of glucocorticoids in hair [19]. In wild felids, the experimental validation of using hair to measure cortisol was less successful [10].

The aim of this study was to evaluate the usefulness of feline hair analysis to assess seasonal changes in cortisol and testosterone levels in four species with different breeding strategies (seasonal and aseasonal breeders).

## 2. Materials and Methods 

### 2.1. Study Site

The study was conducted in 2019–2020 at the Joint Usage Center “Live Collection of Wild Mammals” of the Severtsov Institute of Ecology and Evolution RAS (biological station “Tchernogolovka”), located 50 km northeast of Moscow (56°00′ N, 38°22′ E). The average air temperature in July is +19 °C, and in January it is −11 °C.

### 2.2. Objects

The objects of the study were adult males and females of domestic cats (*Felis catus*), Eurasian lynx (*Lynx lynx*), caracals (*Caracal caracal*) and Amur wildcats (*Prionailurus bengalensis euptilura*). The number and age of the animals are described in Table 1.

### 2.3. Husbandry Conditions

Each cat lived in an outdoor enclosure (for more details, see [1,26,27]), and details are described in Table 2.

The enclosures were located outside, with natural light and temperature. The winter indoor enclosures (for caracal) were located in closed wooden houses where the temperature was kept +14–16° C, over the entire winter and spring. Artificial light was provided under the 12:12 day/night cycle. When the temperature was above −5 °C and deemed safe for the animals, they were allowed into outdoor enclosures.

The animals were fed once a day, 6 days a week, and were fasted 1 day a week. The diet was whole chicken (about 0.3–0.5 kg minced meat per domestic cat and Amur wildcat, and about 1 kg per lynx and caracal). Animals were provided with water ad libitum. In summer, the daily ration of the females during the second part of the pregnancy and the lactating females with kittens (one lynx, two Amur wildcats and 7 domestic cats) was increased by approximately 1.5 times.

### 2.4. Samples Collection

Hair was collected from all individuals four times a year either under anesthesia (Eurasian lynx and caracals) [28] or restraint (Amur wildcats and domestic cats) [1]. Samples were collected in winter from 19 January till 23 February; in spring, 10–18 April (excluding two domestic cats sampled on 3 March 3); in summer, 18 July–24 August; and in autumn, 16 September–10 November. The hair samples (0.1–0.3 g) were collected by cutting hair from the rump of the individual with an electric trimmer at the level of 1 mm from the skin. Then, the samples were transferred to a zip-locked bag by hand using surgical gloves (to exclude the hormones transfer from the hands of the researcher), packed, labeled and kept in the freezer (−20 °C) till extraction.

### 2.5. Steroid Extraction and Analysis

We used the previously described method of steroid extraction with 100% methanol [29]. Briefly, the small amount of hair was washed in 2 mL of 100% methanol and dried, at 55 °C, in the Techne Sample concentrator (Techne FSC400D Sample, Techne Corporation, Minneapolis, MN, USA). Dry hair was chopped into small pieces and weighed in 15 mL tubes with the precision 0.0001 g using an Ohaus Pioneer scale (Ohaus Europe Gmbh, Nanikon, Switzerland). We added 100% methanol to the tube based on the ratio of 10 mL to 0.1 g of hair (for example, if the hair mass was 0.087 g, we added 8.7 mL of methanol). The tubes were rotated for 24 h using a Bio RS-24 mini-rotator (Biosan, Riga, Latvia). Afterwards, 2 mL of the supernatant was transferred to a clean Eppendorf tube and dried by evaporation using a Techne Sample concentrator; the residual sample was reconstituted in 0.4 mL of PBS and stored, at −20 °C, until analysis.

Testosterone (T) and cortisol (C) were measured with ELISA using the commercial kits of Chema (Moscow, Russia). The consistency of extraction was estimated for ten samples collected at the same time, and the CV was 5.5% for the cortisol and 9.4% for testosterone. The cross-reactivity of testosterone antibodies was 16% to 5α- dihydrotestosterone, 1% for androstenediol, and 0.4% for androstenedione, and it was less than 0.1% for all other tested hormones. The cross-reactivity of cortisol antibodies was 5.6% to prednisolone, 0.9% for 11-desoxycortisol, and 0.6% for corticosterone, and it was less than 0.1% for all other tested hormones. The accuracy of both tests was 90–100%. Intra-assay CV was 8.0% for T and 10.0% for C; inter-assay CV was 6.9% for T and 15.5% for C. The sensitivity of the kits was less than 0.006 ng/mL for T and less than 0.04 ng/mL for C.

### 2.6. Statistics

Statistical analysis was performed using the program Statistica 12 (StatSoft Inc., Tulsa, OK, USA). Testosterone and cortisol levels in the total data set and in the domestic cat (as a species) confirmed a normal distribution (Shapiro–Wilk’s W test, *p* > 0.05). Influencing factors on hormonal parameters were evaluated using the parametric statistical method (a general linear model, GLM). We used season (spring, summer, autumn, and winter), species (lynx, caracal, domestic cat, and Amur wildcat) and sex (male and female) as the categorical factors affecting the hormonal level. For post hoc analysis for the domestic cats dataset, we used Student’s *t*-test. To estimate the effect of seasonality on hormone level, we used the Friedman ANOVA test.

## 3. Results

All three factors (species, season and sex) affected the level of testosterone in felids: “species” (F = 14.07; df = 3; *p* = 0.000000), “sex” (F = 17.50; df = 1; *p* = 0.000049), and “season” (F = 11.42; df = 3; *p* = 0.000001). “Species” and “season” also affected cortisol concentrations (F = 14.87; df = 3; *p* = 0.000000, and F = 7.03; df = 3; *p* = 0.000189; respectively). The sex of animals affected cortisol level only as a tendency (F = 3.61; df = 1; *p* = 0.059).

### 3.1. Sex Differences in Testosterone and Cortisol Levels

**Figure 1:**
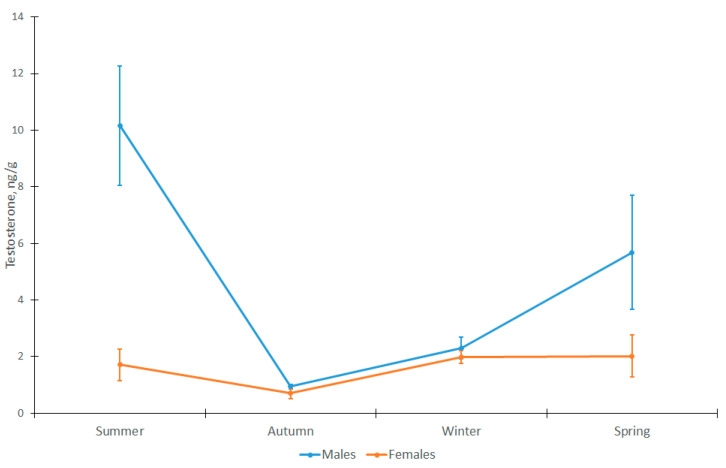
Testosterone concentration in hair of male and female domestic cats (males *n* = 5; females *n* = 12).

In the domestic cat, sex significantly influenced testosterone levels in the hair of the animals (GLM: MS = 141.24; df = 1; F = 19.46; *p* = 0.000041). Significant differences were seen in the spring and summer periods (Student’s *t*-test: n_1_ = 12; n_2_ = 5; t = 2.15; *p* = 0.048 and t = 5.36; *p* = 0.00008; respectively), when the testosterone level was significantly higher in males compared to females (by 2.8 and 5.9 times, respectively) (Figure 1).

In the Amur wildcat, males had higher levels of testosterone than females. The large differences in testosterone concentration were detected only in the spring, when the testosterone concentration in males was 2.8 times higher than in females. In the Eurasian lynx, the same trend was revealed: only in the spring were testosterone levels higher (1.5 times) in males than in females.

In caracals, the level of testosterone in males was higher from autumn to spring, exceeding the level of testosterone in females by 1.8–7.4 times, and it was highest in winter.

**Figure 2:**
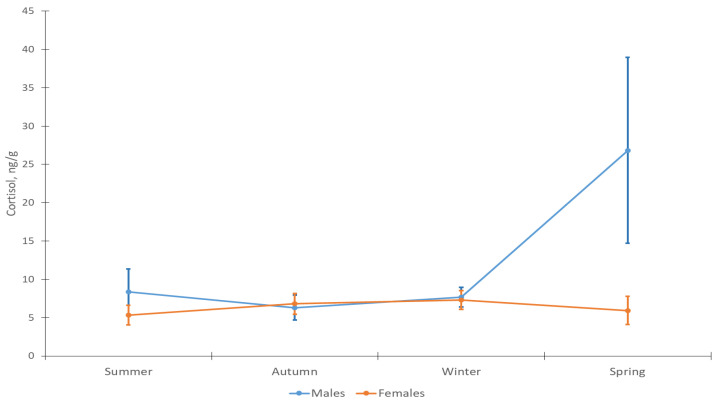
Cortisol concentration in hair of male and female domestic cats (males *n* = 5; females *n* = 12).

In domestic cats, sex affected cortisol only at a trend level (GLM: MS = 269.76; df = 1; F = 3.96; *p* = 0.0509). Significant differences were found in the spring (t = 2.14; n_1 =_ 12; n_2 =_ 5; *p* = 0.00043) (Figure 2), when the level of cortisol was significantly higher in males compared to females (3.6 times).

In the lynx and caracal, there were no differences in the level of cortisol associated with the sex of the animals, in contrast to the Amur wildcat, in which clear sex differences were seen in the winter, when the level of cortisol was significantly higher (3.9 times) in males.

### 3.2. Seasonal Features of the Dynamics of Testosterone and Cortisol in Felines

**Figure 3:**
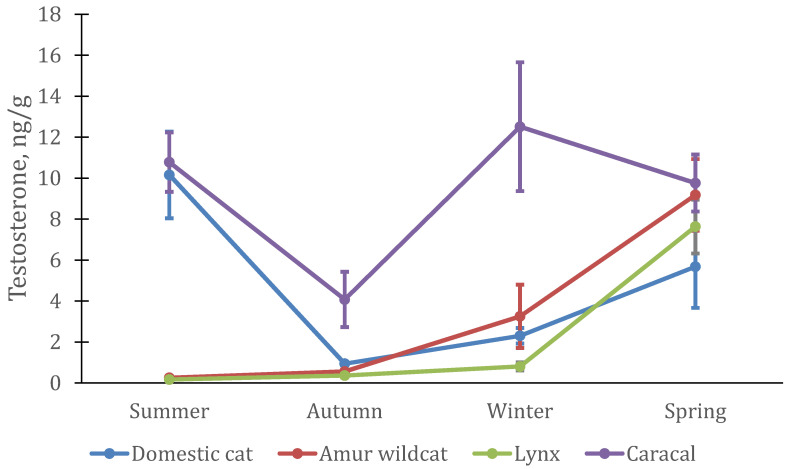
Testosterone concentration in hair from four male felid species (*n* = 5 domestic cats, *n* = 4 lynx, *n* = 4 Amur wildcats, *n* = 4 caracals).

**Figure 4:**
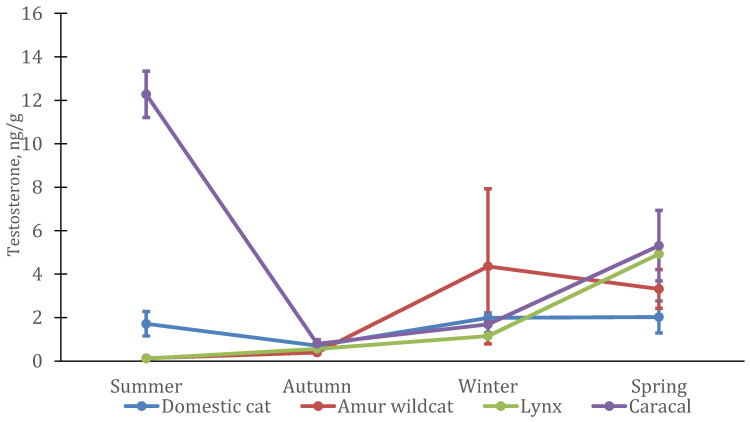
Testosterone concentration in hair from four female felid species (*n* = 12 domestic cats, *n* = 4 lynx, *n* = 4 Amur wildcats, *n* = 2 caracals).

Season had a significant effect on testosterone levels in domestic cats (GLM: MS = 36.19; df = 3; F = 4.98; *p* = 0.0036), primarily because of changes in male levels (F-A: *n* = 5; df = 3; χ^2^ = 13.56; *p* = 0.0036 (Figure 3)). In females, testosterone concentrations remained virtually unchanged throughout the year (F-A: *n* = 12; df = 3; χ^2^ = 5.7; *p* = 0.13 (Figure 4)), whereas in males, it increased sharply in spring (approximately 2.5 times) and then almost doubled in summer.

In males of the Amur wildcat, the testosterone level changed in a similar way to domestic ones (F-A; *n* = 4; df = 3; χ^2^ = 12.00; *p* = 0.0074); however, the increase began in winter, and maximum concentrations of testosterone were reached in the spring. It is interesting that the level of testosterone in females of the Amur wildcat also increased in the winter–spring period (F-A: *n* = 4; df = 3; χ^2^ = 11.1; *p* = 0.011), although in spring it was significantly lower than in males. In male Eurasian lynx, changes in testosterone levels were similar to those in Amur wildcat males (F-A: *n* = 4; df = 3; χ^2^ = 11.1; *p* = 0.011) with a slight increase in the winter period and a subsequent increase (nine times) by April (spring). In lynx females, the pattern of testosterone changes was similar to that of the males: significant changes were observed between seasons. (F-A: *n* = 4; df = 3; χ^2^ = 9.3; *p* = 0.026).

In contrast with the other species, testosterone in caracals showed a different pattern: testosterone levels in males did not change significantly (F-A: *n* = 4; df = 3; χ^2^ = 3.6; *p* = 0.31). It was minimal in autumn, growing strongly towards winter, and remaining practically at the same level both in spring and summer. The maximum level of testosterone was in the spring. In females, testosterone levels were at their highest level in summer.

Changes in cortisol levels in domestic cats were not significant during the year (Figure 2). In females, the average concentration of cortisol remained approximately at the same level (5.3–6.8 ng/g hair) (F-A: *n* = 12; df = 3; χ^2^ = 0.90; = 0.825), while in males it increased in spring (from 6.3–7.6 to 26.8 ng/g hair) (F-A: *n* = 5; df = 3; χ^2^ = 2.04; *p* = 0.56).

**Figure 5:**
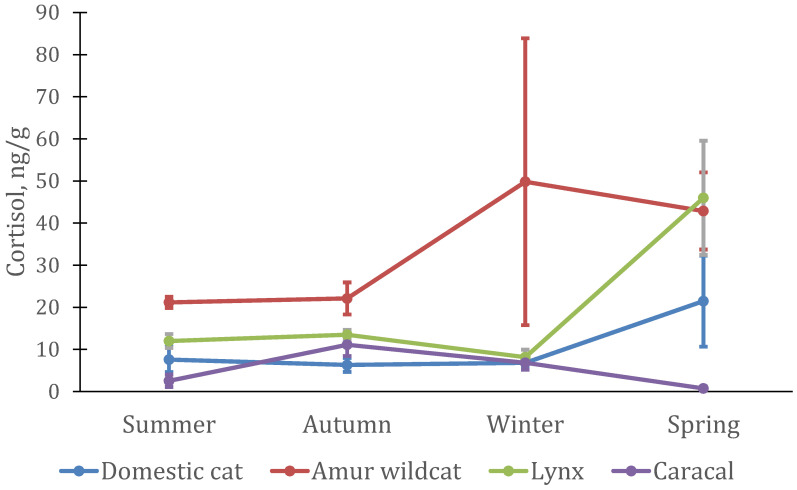
Cortisol concentration in hairs from four male felid species (*n* = 5 domestic cats, *n* = 4 lynx, *n* = 4 Amur wildcats, *n* = 4 caracals).

**Figure 6:**
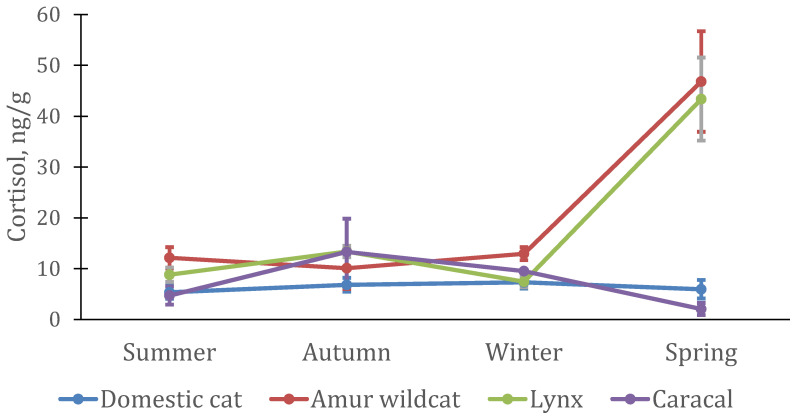
Cortisol concentration in the hair of four female felid species (*n* = 12 domestic cats, *n* = 4 lynx, *n* = 4 Amur wildcats, *n* = 2 caracals).

In males and females of the Amur wildcat, as well as in domestic cats, the level of cortisol did not change significantly during the year (Figure 5). The maximum level of cortisol in males was in the winter–spring period (F-A: *n* = 4; df = 3; χ^2^ = 2.70; *p* = 0.44), while in females, it increased only in spring (F-A: *n* = 4; df = 3; χ^2^ = 5.10; *p* = 0.64) (Figure 6)). A sharp increase in the level of cortisol in the spring period was also noted in Eurasian lynx, both in males (F-A: *n* = 4; df = 3; χ^2^ = 11.1; *p* = 0.011) and in females (F-A: *n* = 4; df = 3; χ^2^ = 10.8; *p* = 0.013). The highest cortisol concentrations in three species of cats (both males and females) occurred in the spring but were not significantly different from concentrations at other times of the year in domestic cats and Amur wildcats, in contrast to Eurasian lynx.

A different pattern of seasonal changes in cortisol concentrations was noted in caracal males (F-A: *n* = 4; df = 3; χ^2^ = 10.2; *p* = 0.017). Cortisol concentrations were at the maximum in animals in the autumn period, decreasing slightly in winter, and the minimum concentrations were in spring and summer. It is interesting to note that the patterns of changes in cortisol levels in male and female caracals were almost identical between the sexes, as in the Eurasian lynx.

### 3.3. Interspecific Features of Testosterone and Cortisol Dynamics in Felines

The average testosterone level and its seasonal dynamics were similar in males of three feline species (domestic cat, Eurasian lynx, and Amur wildcat) with lowest concentrations in autumn, an increase in winter, and highest values in spring. In the Amur wildcat and lynx, testosterone levels dropped sharply in summer, while in male domestic cats, it was even higher than in spring. In caracals, the level of testosterone in the hair was slightly higher than in other felines, the minimum values were also noted in the autumn period. In females, the pattern of changes was similar to that in males (at least in lynx, Amur wildcat and domestic cats), with a maximum level of testosterone in the spring and an earlier rise in the Amur wildcat (as in males) already in winter. In female caracal, the maximum testosterone level was measured in the summer, but, similarly to males, it was higher in spring and winter than in autumn.

Cortisol levels changed almost equally in male and female lynx, male domestic cats, and female Amur wildcats. It significantly increased during the mating period, and in males of Amur wildcats, its increase occurred already in the winter period. In Amur wildcat males, the level of cortisol (21–50 ng/g) was significantly higher than in other felines (0.8–5.3 ng/g) (except for the spring period). A different pattern of cortisol changes was demonstrated by the caracal, in which (in both males and females) the cortisol level was at a maximum in autumn, slightly decreasing in winter, and at a minimum in summer.

## 4. Discussion

The level of testosterone in the hair of domestic cats was significantly higher than that of females only in the spring–summer period. In autumn and winter, the average testosterone levels in males and females were practically the same. A comparative analysis of testosterone levels in conspecifics of different sexes for felines has not been carried out, although our previous data, based on numerous blood samplings in captivity, suggest that sex differences are minimal [30]. Among other predatory mammals, spotted hyenas stand out, whereby the level of androgens in the blood of females is higher than in males [31], and there are similar examples in other orders of mammals, for example, some primates [32].

In temperate climates and in outdoor enclosures, domestic cats showed a fairly clear breeding season (spring–summer), as was previously revealed in our colony [33] and in other published studies from the northern hemisphere [34,35]. Sampling in mid-January reflected hormone levels at the beginning of the year (assuming a two-week time lag between increases in blood and hair hormones [21]), when, apparently, the activity of males’ reproductive system was at the low level based on hormonal data and sperm analysis [33].

A similar trend was noted in the Eurasian lynx and the Amur wildcat, in which sex differences in testosterone levels were noted only during the period of sexual activity of animals—in spring. Eurasian lynx [36] and, to a slightly lesser extent, the Amur wildcat [37] are seasonal breeding species, and they reproduce only once a year. The mating season takes place in late February–early April [36], which is reflected in the collected samples.

In caracals, testosterone levels in males were higher than those in females from autumn to spring, especially in winter. Unlike the other two wild feline species, caracals breed almost all year round [38,39,40]. Males maintain a high level of reproductive activity throughout most of the year. Interestingly, during the summer period, the absence of sex differences in testosterone levels was explained not by a decrease in testosterone levels in males, but by their significant increase in females during this period. Significant changes in androgen or testosterone levels in mammalian females often reflect their physiological status, such as pregnancy [41,42], especially when carrying male embryos [43]; however, in this study, the females were not mated with the male. Significant increases in androgen levels, including testosterone, in the ovaries of mammalian females may also occur during the last stage of proestrus [44], as precursors of estradiol; there is also an increase in testosterone in the blood during estrus. Its increase in the ovaries suggests a tractable increase of it in the blood [45]. It is possible that the increase in the level of testosterone in the hair of female caracals was associated with the active cycling of females in the summer.

Cortisol levels did not differ significantly between male and female felines, although small sample sizes often prevented statistical analysis of the data. Gender differences in the level of glucocorticoids have been repeatedly noted in various groups of mammals: ungulates [46], primates [47] and carnivores [48,49], including felids [50]. Significant individual changes in the level of cortisol often overlapped the sex differences in these animals, in particular, in winter, whereby in some males of the Amur wildcat, the level of hormones differed by more than 15 times (see also [37,51]). In a relatively large data set of domestic cats, it was shown that mean cortisol levels in males were significantly higher than in females during the spring. These differences were explained by the conditions in which the animals were kept. Although domestic cats do not commonly show increased stress when kept in small groups [52], the total number of cats at the Tchernogolovka station varied from 45 to 55 during the year. Some females were in estrus, which in felines is associated with an increase in acoustic and marking activity [53]. In the period March–April, males were involved in mating, spending a lot of time “guarding” the female (including the females in neighboring enclosures), showing mating and courtship activities, and reducing feeding activity. All these mating–related changes could have led to increases in cortisol levels in males.

The seasonal changes in testosterone in the hair of male felines reflected the seasonality of reproduction of specific species described in the literature. In seasonal breeding species, the Eurasian lynx [36] and the Amur wildcat [37], the peak of testosterone levels in males occurred in the spring, i.e., almost coincided with the mating period (in other mammals it coincides with the increase in Sertoli cell number at testicular level [54,55]). For lynx, this correlates with the hormonal data obtained by measuring hormones in male feces [56]. In domestic cats it remained high even in summer. In central Russia, cats mate almost until the end of August [33]. In males of all these three species, an increase in testosterone levels began already in winter, before the mating season. Gradual increases in testosterone levels and associated spermatogenesis [36] start before the beginning of the mating season. In caracal, a species with non-seasonal breeding [38], a relatively high level of testosterone persisted throughout almost the entire year, decreasing slightly in autumn. It is possible that the light period (shortening of daylight hours) had a negative effect on the activity of the reproductive system of caracals in the autumn as described, for example, for Pallas’ cat [57].

Interestingly, mean testosterone levels in female felines changed in a similar manner to those of males. In lynx and Amur wildcats, the level of testosterone increased significantly by the spring. In part, this may be due to the need to regulate the sexual behavior of females during this period and the participation of testosterone as an intermediate substance in the production of estradiol [44]. At the same time, an increase in testosterone levels in female felines during pregnancy can also be assumed [41,42,43], which began in March in Amur wildcats and lynx. In caracal females, some increase was noted in the summer, while in domestic cat females, there were no significant seasonal changes in testosterone levels.

The seasonal dynamics of cortisol in different species also differed. The clearest pattern was observed in the Eurasian lynx, where changes in cortisol levels in males and females occurred almost synchronously and showed a significant increase only during the mating season. In females and males of the Amur wildcats, the level of cortisol also increased in spring, although in males, an increase was noted already in winter. In male domestic cats, cortisol levels also peak in spring (but not in females). Apparently, the main reasons for the increase in cortisol levels are the activation of the HPA system in animals during the mating period, which is associated with an abundance of acoustic, visual, and olfactory stimuli in the conditions of the breeding center. The presence of sex differences in the patterns of changes in cortisol concentrations may also indicate a greater activity of males during the mating season, which has been repeatedly noted in felines, including domestic cats [58] and lynx [59].

It is interesting to note that the maximum levels of cortisol in Amur wildcats were previously recorded in winter [37]. Indeed, low air temperatures require an increase in metabolic intensity in animals to maintain a constant body temperature; a negative correlation between ambient air temperature and glucocorticoid levels was also found for other felines, for example, the Amur tiger (*Panthera tigris altaica*) [60,61]. Obviously, low air temperatures in winter can have a negative impact on the level of glucocorticoids, especially in tropical species, in particular the Amur wildcat as a subspecies of the Bengal cat. However, the winter of 2019–2020 was extremely warm, and the average January temperature was −0.4 °C, which is more than 10 degrees above the norm (www.rp5.ru, 4 February 2022). It is likely that at such relatively high temperatures, the increase in cortisol levels in most animal species was negligible.

A completely different pattern of changes in the level of cortisol was observed in males and females of caracals (almost synchronous), which was probably associated with changes in the conditions in which the animals were kept during the year. The maximum level of cortisol was noted in the animals in autumn, when the animals were kept in outdoor enclosures, and nighttime temperatures regularly dropped below zero degrees. In winter, the animals were kept in very small (4 m^2^) indoor cages and close enough to each other, without access to the outdoor enclosures, which were being reconstructed at that moment. Starting in the spring, all animals had access to external enclosures and an internal warm room; in summer, some of the animals were transferred to open enclosures. Taking into account that for another tropical felid, the tiger [61], a negative correlation was found between the level of cortisol and ambient temperature, which was probably due to an increase in the energy consumption of animals to maintain body temperature, an increase in the level of cortisol in caracals in autumn is quite expected. At the same time, in winter, the main determining factors could be the small size of the enclosure and the presence of con- and heterospecifics, as described, for example, for clouded leopards (*Neofelis nebulosa*) [62] and cheetahs (*Acinonyx jubatus*) [50].

A comparison of the level of hormones in the blood of animals does not always adequately reflect their real significance for certain organisms. In particular, the effect of hormones on target tissues can be largely determined by the concentration of hormone receptors [63], and not by the concentration of hormones in the blood itself. A comparison of hormone levels in excreta is even more difficult, as the proportion of hormones excreted in urine and feces differs significantly between species [64,65,66]. How much the hormone concentrations in hair correspond to those in the blood in different mammalian species is still an open question; however, our studies of the seasonal dynamics of testosterone and cortisol show their correspondence to those described in the blood of animals or expected patterns based on the characteristics of the reproductive biology of a particular species. In this situation, we also conducted cross-species comparisons, realizing that these results should be treated with a certain degree of caution.

Mean hair testosterone levels in male felines ranged from 0.16 to 12.51 ng/g. A similar trend was shown in lynx and Amur wildcats, as well as domestic cats (except for the summer period). The main changes in hormone concentrations were related directly to the onset of the mating period in felines (spring in all three species). In domestic cats, the mating season was significantly extended and high testosterone levels were recorded in the summer. In caracals, the average testosterone level did not fall below 4.07 ng/g of hair even in autumn, which indicates the almost year-round activity of the reproductive system in this feline species, even in central Russia. It should be noted that this level of testosterone almost corresponded to the level of testosterone in other feline species during the mating season. Year-round reproduction of the caracal in the wild confirms this hypothesis [38]. How comparable it is, considering interspecies differences in blood testosterone levels, is not yet clear and requires further measurements; a comparative analysis of data from different laboratories is most likely incorrect due to differences in antibodies used in different test systems.

In females, changes in testosterone levels only showed a slight increase during the spring, which could be associated with the manifestation of sexual behavior in females in that time. In addition, some females (lynx, Amur wildcats and domestic cats) were pregnant at that point. These processes are associated with increased levels of androgens in a number of mammalian species [41,42,43,44], possibly also in cats. In female caracal, a sharp increase in testosterone levels was witnessed in the summer. The reasons for this increase are not clear, but unlike most other felines, female caracal came into estrus in the summer, and testosterone levels may have increased during the last stage of proestrus [44], as precursors of estradiol.

Other patterns of changes in cortisol levels in animals have been noted as well. In particular, a sharp increase in cortisol levels in all seasonally breeding cat species (lynx, Amur wildcat and domestic cat) was noted in all males during and after the mating season (spring). In the Amur wildcat, this increase began already in winter, but in general, in males of this species, the level of cortisol in the hair was almost twice as high as in males of other species. The dramatic increase in hair cortisol levels in spring was likely due to the mating season (increased activity, response to multiple visual, acoustic, and olfactory stimuli). Only seasonal changes in cortisol levels in caracals did not match those in other felines.

In female Amur wildcats and Eurasian lynx, a sharp increase in cortisol levels was also noted in the spring period (the mating period and the first trimester of pregnancy). Their cortisol concentrations were similar to those of males. During these periods, their concentrations were significantly higher than in domestic cats and caracals, as well as during most of the year (differences are not significant). The results from our laboratory show that domestic cats generally have lower serum cortisol levels than wild felines (lynx and Amur forest cat) [30]. This is consistent with the results obtained in the analysis of hair, in particular, higher levels of cortisol in lynx and Amur wildcats than in domestic cats during the mating season (and for Amur wildcats in winter). The caracals used in the present study lived as in-house pets for several years before they came to the station, and were accustomed to the presence of humans, which could explain some of the increased “stress resistance” of these animals.

Thus, changes in the concentrations of testosterone and cortisol in the hair of four feline species adequately reflect the dynamics of the annual reproductive cycle for each of the species (testosterone), and are in good agreement with the husbandry characteristics of animals in captivity during the year. This makes it possible to use this indicator to analyze the condition of animals in captivity and in the wild, including by non-invasive methods, which is essential for the study and conservation of endangered species.

## 5. Conclusions

Hair testosterone levels in four felid species coincided with their breeding traits: increased during the mating season in seasonal breeders, stayed high in aseasonal caracal, and showed higher concentrations in males than in females. Hair cortisol level also demonstrated a valid pattern: changes in husbandry conditions (smaller cages) resulted in an increase in cortisol; mating period and animal excitement were also related with the increase in cortisol. The measurement of these two hormones in hair samples of felids may be a reliable tool for the monitoring of hormonal changes in individuals in captivity and in the wild.

## Figures and Tables

**Table 1:** Number of experimental animals.

Species	Number of Males	Number of Females	Age Limits (Years)
Domestic cat	5	12	1–6
Eurasian lynx	4	4	3–12
Caracal	4	2	2–9
Amur wildcat	4	4	2–7

**Table 2:** The traits of animals’ husbandry conditions.

Species	Type of Enclosure	Size, m^2^
Domestic cat	Outdoor	4–6
Eurasian lynx	Outdoor	74
Caracal (summer–autumn)	Outdoor	12–74
Caracal (winter–spring)	Indoor (+15 °C) with regular walking hours in outdoor enclosure	4 (indoor) + 12 (outdoor)
Amur wildcat	Outdoor	8–16

## Data Availability

The hormones’ (testosterone and cortisol) concentrations at all points in each animal may be provided by the corresponding author (snaidenko@mail.ru) upon request.
